# Down-Shifting in the YAM: Ce^3+^ + Yb^3+^ System for Solar Cells

**DOI:** 10.3390/ma14112753

**Published:** 2021-05-23

**Authors:** Bartosz Fetliński, Sebastian Turczyński, Michał Malinowski, Paweł Szczepański

**Affiliations:** 1 Institute of Microelectronics and Optoelectronics, Faculty of Electronics and Information Technology, Warsaw University of Technology, 00-662 Warsaw, Poland; m.malinowski@elka.pw.edu.pl (M.M.); p.szczepanski@elka.pw.edu.pl (P.S.); 2 Sieć Badawcza Łukasiewicz—Instytut Mikroelektroniki i Fotoniki, 02-668 Warsaw, Poland; seb.turczyn@gmail.com

**Keywords:** Y_4_Al_2_O_9_ crystal, energy transfer, Ce^3+^ ion, Yb^3+^ ion, solar spectrum conversion, photovoltaic cells

## Abstract

In this work, we investigate Ce^3+^ to Yb^3+^ energy transfer in Y_4_Al_2_O_9_ (YAM) for potential application in solar spectrum down-converting layers for photovoltaic devices. Photoluminescence properties set, of 10 samples, of the YAM host activated with Ce^3+^ and Yb^3+^ with varying concentrations are presented, and the Ce^3+^ to Yb^3+^ energy transfer is proven. Measurement of highly non-exponential luminescence decays of Ce^3+^ 5d band allowed for the calculation of maximal theoretical quantum efficiency, of the expected down-conversion process, equal to 123%. Measurements of Yb^3+^ emission intensity, in the function of excitation power, confirmed the predominantly single-photon downshifting character of Ce^3+^ to Yb^3+^ energy transfer. Favorable location of the Ce^3+^ 5d bands in YAM makes this system a great candidate for down-converting, and down-shifting, luminescent layers for photovoltaics.

## 1. Introduction

One of the methods considered for improving the efficiency of photovoltaic (PV) devices, beyond the single-junction cell limit, is a conversion of the solar irradiation spectrum before it reaches the cell’s surface. From a photovoltaic applications standpoint, the down-conversion and down-shifting processes are particularly interesting for the conversion of diffused radiation, with more short-wavelength content. The enhanced efficiency of photovoltaic devices, in the 300–500 nm spectral range, would improve the performance of devices operating under diffused solar irradiation. This feature makes the idea of solar spectrum converters particularly important for off-grid systems, as it would improve their performance under overcast and low irradiance conditions, improving their reliability and decreasing the probability of loss of load [1].

The Ce^3+^ + Yb^3+^ pair is often considered for down-conversion of the solar spectrum for photovoltaic applications. In the case of down-conversion, a two-photon process, a twofold increase of photogenerated current for the absorbed spectral range is possible, even assuming identical external quantum efficiency (EQE) of a solar cell in the spectral range of both absorption and emission. The EQE is defined as a ratio of a number of carriers participating in the photocurrent and number of photons reaching the surface of a photovoltaic device. The EQE is commonly used to describe spectral properties of photovoltaic devices, as it combines effects of the internal structure of a PV device, material properties, as well as reflection. Down-shifting, a single photon process, brings any benefit for a photovoltaic device only if EQE is larger in the emission spectral range than in the absorption band, as only then a higher photocurrent may be obtained. The main advantage of Ce^3+^ + Yb^3+^ system, in comparison with other rare-earth ions pairs such as Tb^3+^ + Yb^3+^ [2] or Pr^3+^ + Yb^3+^ [3], is broad and tunable in UV, and partially VIS, absorption range of the Ce^3+^ ions. Narrow absorption peaks, resulting from f-f transitions in the rare earth ions, are arguably the most important factor preventing the obtaining of efficient, and practical, down-converter for photovoltaic devices. In order to obtain efficient excitation of Yb^3+^ via efficient down-conversion process Bi^3+^ is also often considered [4,5]. The location of Ce^3+^ absorption bands is very heavily influenced by the host matrix used, which allows it to fine-tune the Ce^3+^ absorption and emission properties to the desired optimum for a given application [6].

In order to be considered for a viable solar spectrum down-converter, the host material for the Ce^3+^ + Yb^3+^ system needs to fulfill two basic requirements. The 5d absorption bands, of the cerium in this host, should be located in the spectral range of 300–500 nm in order to absorb a significant share of energy carried by solar irradiation. On the other hand, the emissions from the 5d cerium bands should have energy, at least, twice that of the Yb^3+^ absorption bands to allow for the down-conversion to take place [7,8,9]. The broad width of the Ce ^3+^ 5d absorption band, along with a significant Stokes shift, introduces a trade-off between these two requirements [6]. From this point of view Yttrium Aluminum Monoclinic (YAM—Y_4_Al_2_O_9_) is known to fulfill requirements for Ce^3+^ host material very well, with favorably located 5d bands’ absorption and emission ranges in this host [10,11]. This makes YAM an attractive candidate host material from the point of view of further down-conversion to Yb^3+^ ions, but also for down-shifting in the YAM: Ce^3+^ system, which may be beneficial for some solar cells.

There are reports of efficient down-conversion in Ce^3+^ + Yb^3+^ systems, reaching 194% [12,13,14,15]. These efficiencies, calculated based on the decrease of Ce^3+^ 5d luminescence decay times, should be treated as absolute maximum due to assumptions commonly made at these calculations. Measurements conducted with other methods, taking into account possible parasitic processes, consistently show smaller efficiencies [16]. In the case of many among the investigated hosts, no cooperative down-conversion from Ce^3+^ to Yb^3+^ had been observed, and single-photon energy transfer from cerium to ytterbium ions was attributed to charge transfer [16,17].

Some functional down-converting layers utilizing the Ce^3+^ + Yb^3+^ pair had been reported [18], along with spectra converting layers, based on other rare earth systems such as SiN:Tb**^3+^** + Yb**^3+^** [19], for c-Si cells or organic dyes [20]. However, obtaining such a layer poses significant challenges, such as reabsorption within Yb^3+^ ions [21] or sub optimally located absorption bands [19].

In this work, we investigate optical properties of the YAM, activated with Ce^3+^ and Yb^3+^, with such potential applications as solar spectrum converting phosphor for photovoltaic applications. A set of samples prepared, using the micro pulling down method, has been characterized using XRD. We present results of characterization of the optical properties of YAM: Ce^3+^ + Yb^3+,^ conducted by measurements of transmission, excitation, and emission spectra. Further, in order to investigate the efficiency of the energy transfer from Ce^3+^ to Yb^3+,^ we present results of luminescence decay time measurements, calculated energy transfer efficiencies, and emission intensity, measured as a function of the excitation power. Finally, we conclude with a brief discussion of observed properties of this system from the point of view of potential use as solar spectra converting material for photovoltaic applications.

## 2. Materials and Methods

The samples used in this work were prepared at the Institute of Electronic Materials Technology (ITME) in Warsaw, Poland. The institute had developed the micro pulling down method for obtaining the polycrystalline YAM samples. The use of this method allows for avoiding problems with cracking the samples during the cooling period, which is a problem when the Czochralski method is used. This is a particular problem for YAM preparation as it undergoes a phase transition at 1300 °C. The micro pulling down was originally developed for the preparation of single-crystal fibers. Obtained YAM samples had a form of polycrystalline rods with a diameter of about 3 mm cut into ~5 mm segments. The sample set consisted of eight samples with 0.5% Ce^3+^ co-doped with 0.1%, 1%, 2% and 5% Yb^3+^, pulled with speed of either 1 mm/min or 5 mm/min. Additionally YAM: 0.2% Ce^3+^ had been used for absorption measurements and YAM: 5% Yb^3+^, YAM: 0.5% Ce^3+^ had been used as a reference. The samples are listed in the Table 1.

The spectroscopic characterization of the samples had been performed using the Photon Technology International (PTI) setup (Birmingham, NJ, USA), consisting of a xenon light source and monochromators. Hamamatsu PMTs type R928 and H10330B-75 (Hamamatsu Photonics K.K., Hamamatsu, Japan) were used as detectors for UV/VIS and NIR ranges, respectively. The absorption measurement had been conducted using Perkin Elmer Lambda 950 spectrophotometer (PerkinElmer Inc., Waltham, MA, USA). Output to input power ratio had been measured using a temperature-controlled laser diode and Thorlabs PM100D power meter (Thorlabs Inc., Newton, NJ, USA). The very fast luminescence decay times of 5d bands of Ce^3+^ had been measured at Warsaw University at CENT Material Technologies Laboratory using a Micro Time 200 Time Resolved Fluorescence Microscope (PicoQuant, Berlin, Germany), with a picosecond laser diode emitting at 402 nm. The wide spectral range of the detector had been limited with a band-pass filter, transparent in 500 nm ± 20 nm range. All the measurements had been conducted at room temperature.

## 3. Results and Discussion

**Figure 1:**
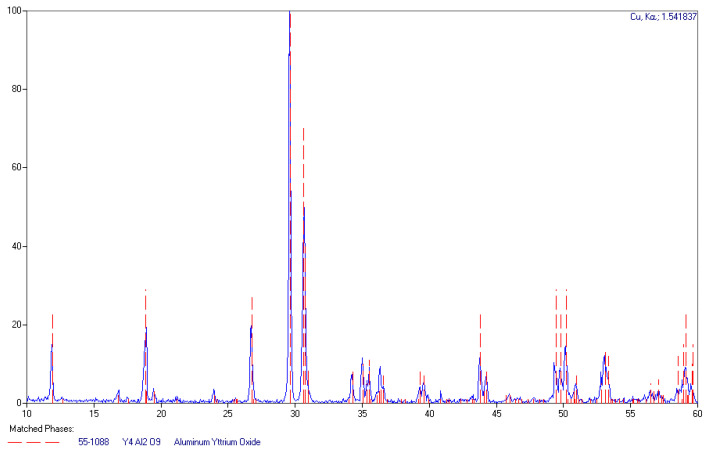
The XRD spectrum of an undoped YAM sample (blue line) and Y_4_Al_2_O_9_ features from the database (red dashed lines).

X-ray diffraction spectra of the selected samples, among those fabricated, were measured to evaluate the correctness of the process and check for the presence of possible unwanted phases. The measured XRD spectrum, presented in Figure 1, corresponds well with features attributed to Y_4_Al_2_O_9_ (YAM) in the database.

**Figure 2:**
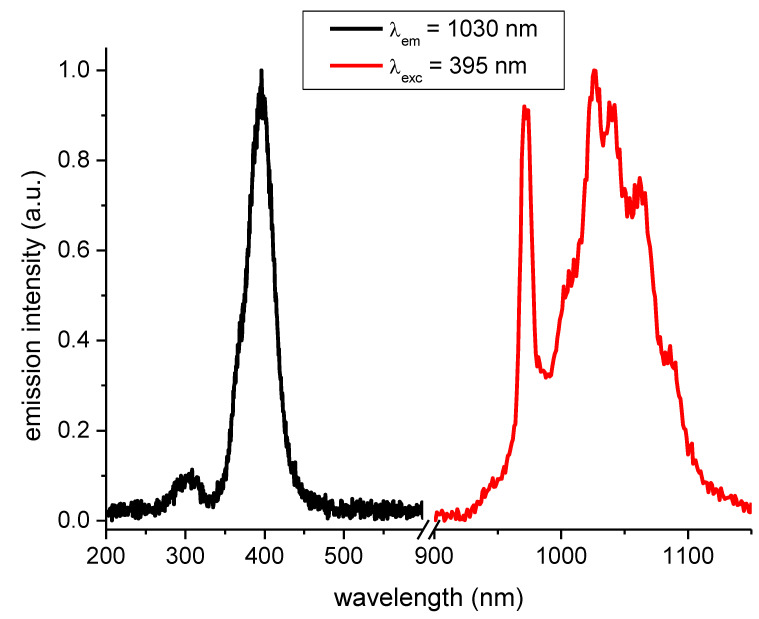
Emission spectrum of Yb^3+^ after Ce^3+^ excitation at 395 nm and excitation spectrum of Yb^3+^ emission monitored at 1030 nm in YAM: 0.5% Ce^3+^ + 1% Yb^3+^ sample.

**Figure 3:**
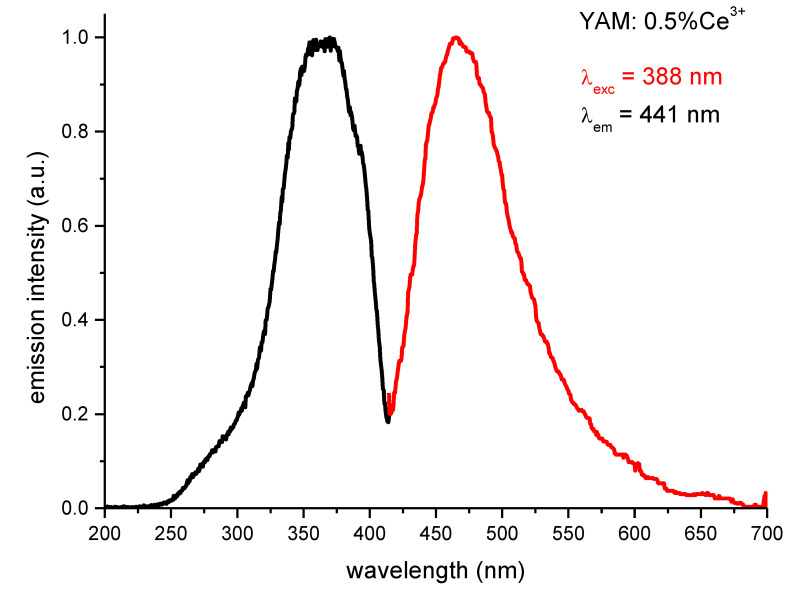
Excitation and emission spectra of Ce^3+^-only activated YAM samples.

Locations of the lower-lying 5d bands of Ce^3+^ the absorption spectrum of thin YAM: 0.2% Ce^3+^ sample had been previously measured and established at 350 nm, 370 nm, and 390 nm [10] in general agreement with a more detailed study [11]. Photoluminescence spectra of the YAM: 0.5% Ce^3+^ + 1% Yb^3+^ system, confirming the existence of a strong energy transfer mechanism from Ce^3+^ to Yb^3+,^ are presented in Figure 2. The excitation spectrum of the Yb^3+^ emission shows a feature at around 300 nm and a broader band centered at 400 nm. These features correspond well to 5d absorption bands of cerium. These features correspond well with the excitation spectrum of YAM activated with Ce^3+^ ions only, as presented in Figure 3 along with cerium emission spectrum.

**Figure 4:**
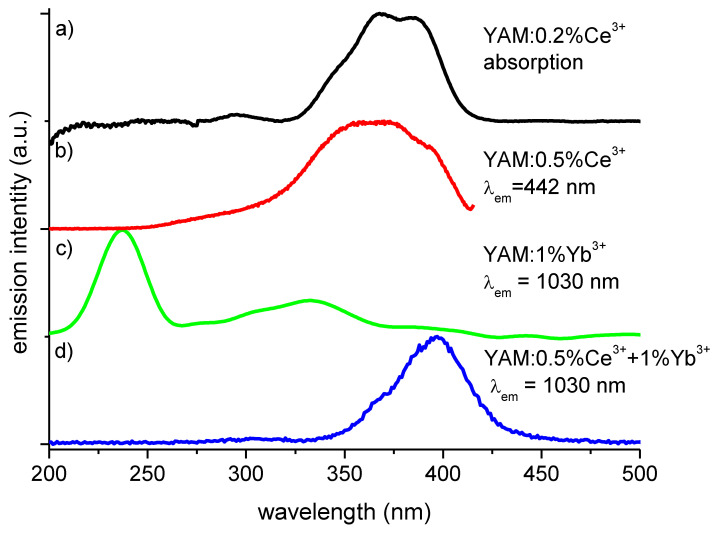
Absorption and excitation spectrum of cerium activated YAM sample (**a**,**b**), the excitation spectrum of Yb^3+^ emission of Yb^3+^ doped YAM sample in the UV/VIS range (**c**), the excitation spectrum of Yb^3+^ emission at 1030 nm of Ce^3+^-Yb^3+^ co-doped sample (**d**).

The 5d bands of Ce^3+^ in YAM are located particularly favorably for down-conversion for solar cells. The energy of the lower 5d^1^ of Ce^3+^ at about 25,200 cm^−1^ (which corresponds to the wavelength of 397 nm) gives a chance of two-photon cooperative down-conversion to two Yb^3+^ ions (^2^F_5/2_ levels with the energy of 10,000 cm^−1^ or approximately 1 µm) to take place. This location of the 5d band also allows for absorption of part of solar spectral irradiance, which carries a useful amount of energy, but is also inefficiently used by a typical crystalline silicon solar cell. The scheme of the ions’ energy levels, and the phenomena resulting in radiative recombination, is shown in Figure 4.

However, the presence of 300–400 nm bands in the excitation spectra of the Yb^3+^ infrared emission is not necessarily proof of energy transfer from Ce^3+^ to Yb^3+^, as the presence of 5d or charge transfer bands of Yb^3+^ is possible, and expected, in that spectral range [22,23]. Figure 3 shows largely overlapping excitation and emission spectra of YAM samples singly-doped with either Yb^3+^ or Ce^3+^ ions, which also simultaneously show different character.

Figure 4 presents absorption spectrum of 1.1 mm thick YAM: 0.2% Ce^3+^ sample (after subtraction from the absorption spectrum of a pure YAM sample) (a) as well as matching excitation spectrum of YAM: 0.5% Ce^3+^ emission observed at 440 nm (b), both exhibiting features at 395 nm, 365 nm, 350 nm, and at around 300 nm. These spectra differ significantly, relative to the excitation spectrum of Yb^3+^—only activated YAM sample in this spectral range (c). The excitation of doubly activated YAM: 0.5% Ce^3+^ + 1% Yb^3+^, with a distinct peak at around 400 nm and a smaller feature at around 300 nm, is consistent with the Ce^3+^ excitation spectrum. The similarity of the excitation spectrum of 1030 nm in the double activated sample, (d) single doped absorption, and excitation spectra (a), (b) suggests that the Yb^3+^ emission is, indeed, primarily excited by Ce^3+^ ions and not Yb^3+^.

**Figure 5:**
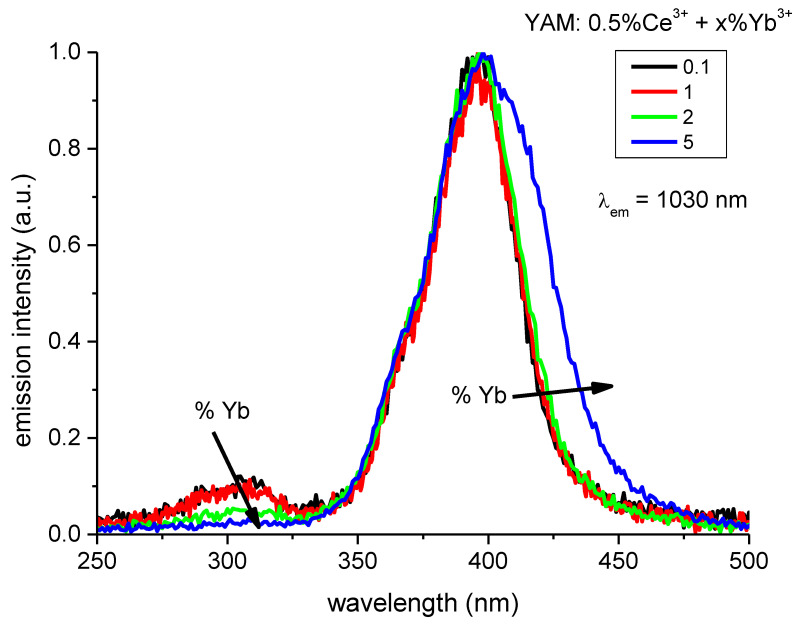
Excitation spectra of Yb^3+^ emission monitored at 1030 nm. With the increased concentration of Yb^3+^ the relative intensity of peak attributed to Ce^3+^ at about 300 nm decreases.

Figure 5 shows the excitation spectra of Yb^3+^ emissions, observed at 1030 nm, in a set YAM: 0.5% Ce^3+^ samples co-doped with varying amounts of Yb^3+^ in 0.1–5% range. The excitation spectra consist of two distinct features at 300 nm and a stronger one with the maximum at 395–400 nm, to which the displayed spectra are normalized. With increasing concentration of Yb^3+^ ions, the intensity of 300 nm feature decreases, up to almost complete disappearance for the 5% Yb^3+^ sample. This effect is a result of an additional pathway, introduced by ytterbium ions, exhibiting a rather weak excitation around 300 nm, which leads to emission in the visible range instead of the desired ^2^F_5/2_ → ^2^F_7/2_ infrared emission. The increased concentration of Yb^3+^ also shifts the slope of the main feature around 400 nm toward longer wavelengths, similarly to the effect reported for YAG [23], although for a wider concentration range.

**Figure 6:**
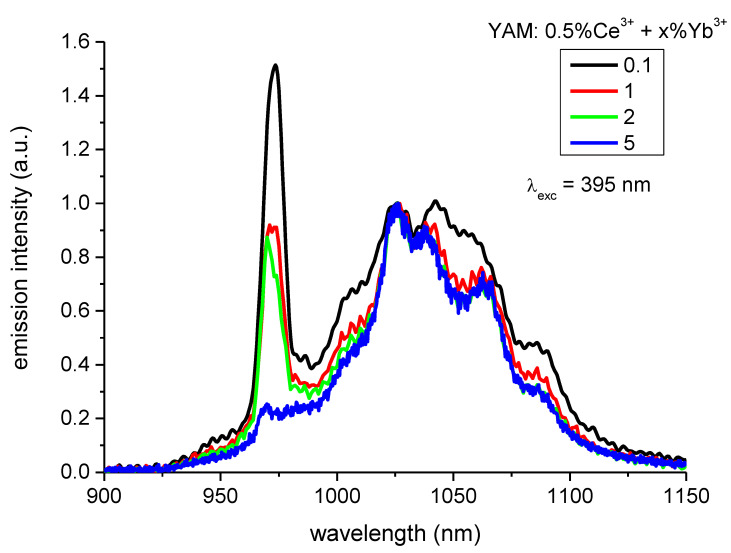
Emissions in the NIR range of Yb^3+^ ions excited at 395 nm. With increased Yb^3+^ concentration the relative intensity of peak at about 980 nm decreases due to reabsorption.

Figure 6 shows the emission spectra of the YAM: 0.5% Ce^3+^ in the 900–1150 nm NIR range for various concentrations of the Yb^3+^ ions co-dopants. The spectra are normalized to emission intensity at 1030 nm. Increasing concentration of Yb^3+^ ions weakens the emission peak at 973 nm, due to reabsorption caused by the partial overlap of this peak emission and Yb^3+^ excitation. While the most beneficial shape of the Yb^3+^ emission is obtained for its lowest concentration of just 0.1% it does not necessarily mean that this would be the optimal concentration for the solar modifying layer of a solar cell, as the trade-off between spectral mismatch, and the absorbance of such a layer, also depends on the layers, geometry, and properties of the considered solar cell.

**Figure 7:**
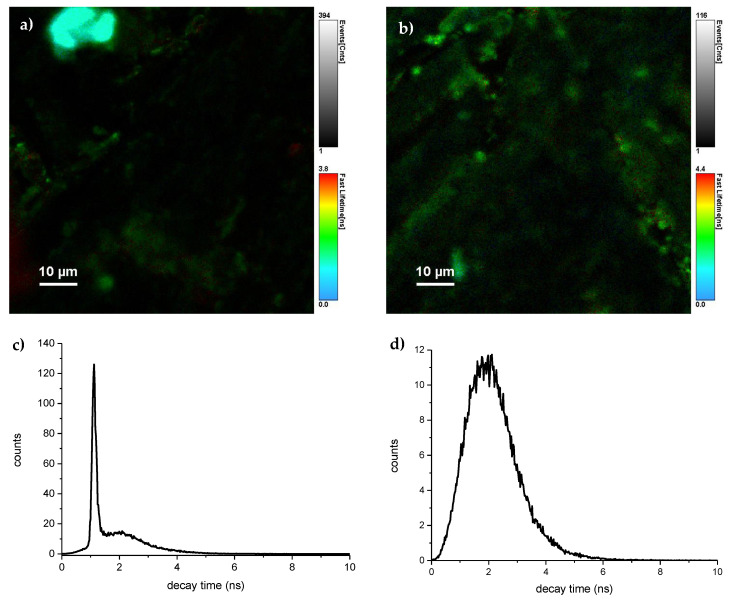
Emission intensity of selected YAM: 0.5% Ce^3+^ + 1% Yb^3+^ sample, in two distinct areas (**a**,**b**). Colors correspond to the luminescence decay times scale. Uniformity of the luminescence decay times is illustrated with histograms of the decay times recorded in the areas pictured above them (**c**,**d**).

To investigate the efficiency of the Ce^3+^ to Yb^3+^ energy transfer, the luminescence decays of cerium ions in samples with varying Yb^3+^ content were measured. The samples had been excited at 402 nm using a picosecond laser diode, at wavelength corresponding to the excitation band presented in Figure 5. Ce^3+^ emission was monitored in 500 ± 20 nm range. The Micro Time 200 Time-Resolved Fluorescence Microscope allowed for luminescence decay measurements with fine spatial resolution. The luminescence intensity, along with measured luminescence decay times, for two distinct areas of the YAM: 0.5% Ce^3+^ + 1% Yb^3+^ is shown in Figure 7. The histograms below show the distribution of the decay times. At the level of magnification used in the measurements, the crystalline YAM: Ce^3+^ + Yb^3+^ shows areas with varying emission intensity. The observed luminescence decay times in the whole investigated areas tend to be described by compact histograms. In some cases (such as the left image in Figure 7), an area with significantly shorter luminescence decay lifetimes, implying higher local concentration of Ce^3+^ ions, was observed. Measurements from areas exhibiting such a distorted histogram were dismissed and retaken in another part of a given sample. For further analysis, decay profiles from high luminescence areas, with indicated lifetimes, close to the histogram, maxima were chosen.

**Figure 8:**
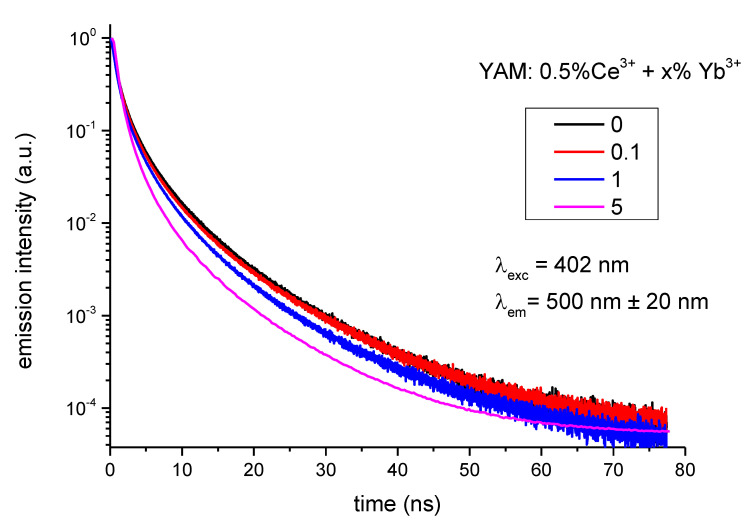
Luminescence decay profiles for samples with varying Yb^3+^ concentration.

The luminescence decay profiles collected for samples with different Yb^3+^ are presented in Figure 8. The profiles were normalized to unity. The Ce^3+^ 5d luminescence decay times in YAM were reported under different excitations and temperatures [10,11,22]. The luminescence decay profiles do not follow single exponential decay; it is also not possible to satisfactorily approximate them with double or triple exponential decay. To calculate energy transfer efficiency the effective luminescence decay times, defined as *τ*_eff_ = ʃ *I*(*t*) *dt,* were used instead. On this basis, the efficiency of the cooperative process is obtained from the luminescence decay profiles of the Ce^3+^ 5d band was calculated using the following equation:

(1) $$\eta_{CET}=1-\frac{\int^{\;}I_{x}dt}{\int^{\;}I_{0}dt}$$

Quantum efficiency (or quantum yield) of the energy transfer process may be calculated assuming only radiative relaxation of excited Ce^3+^ and Yb^3+^ ions (*η*_Ce_ and *η*_Yb_ equal to 1):

(2) $$\eta_{QE}=\eta_{Ce}\left(1-\eta_{CET}\right)+2\eta_{Yb}\eta_{CET}$$

**Figure 9:**
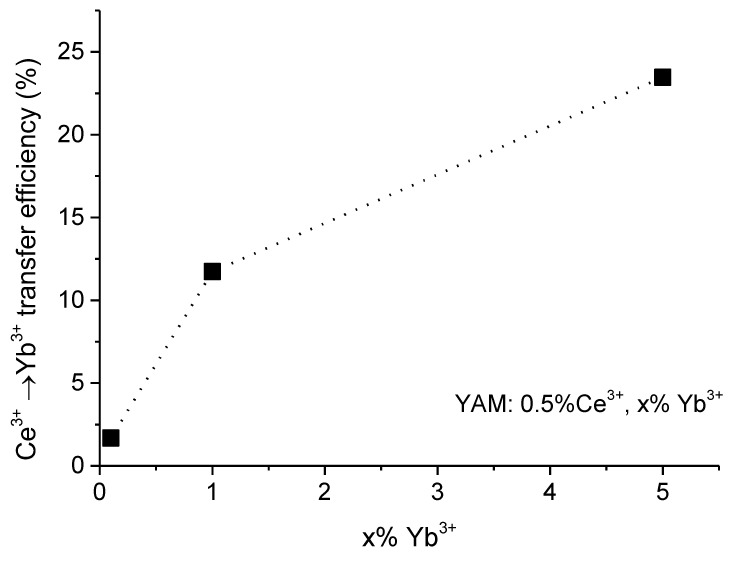
Calculated Ce^3+^ → Yb^3+^ energy transfer efficiency.

Maximal quantum efficiency, estimated using this method, is 123% for the YAM: 0.5% Ce^3+^ + 5% Yb^3+^ sample. Values calculated for different Yb^3+^ concentrations are presented in Figure 9 Given the assumptions mentioned above this obtained value should be treated as an absolute theoretical maximum of this process. Even with these moderate assumptions, the observed quantum efficiency is low if the down-conversion process is, indeed, the mechanism of energy transfer.

**Figure 10:**
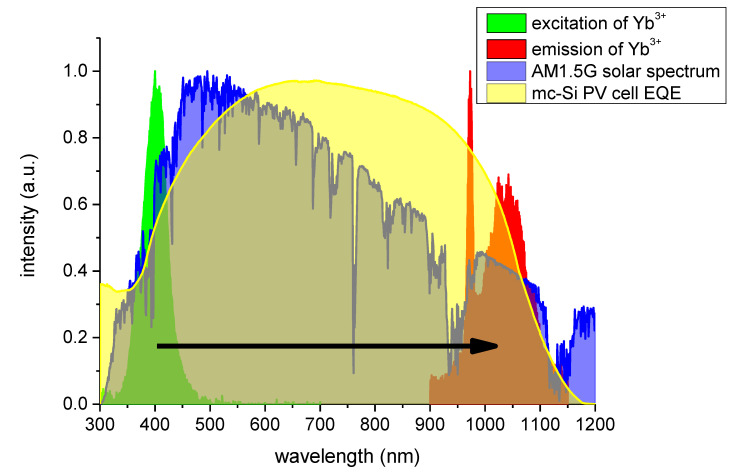
Excitation and emission bands of YAM: Ce^3+^ + Yb^3+^ phosphors overlaid on AM1.5G solar spectrum and EQE of a typical crystalline solar cell.

The efficiency of this process increases with rising Yb^3+^ ions concentration. However, for photovoltaic applications, it should be remembered that the benefits of higher efficiency, of Ce^3+^ → Yb^3+^ energy transfer, would be, at least, partially offset due to a relatively decreased emission of Yb^3+^ ions at 973 nm, as shown in Figure 6. While the overall emission from Yb^3+^ may increase with its concentration, the emission in the range of 1000–1070 nm, which would also become stronger with increasing Yb^3+^ concentration, is generally more mismatched with an external quantum efficiency of a typical crystalline silicon-based solar cell, as demonstrated in Figure 10. This effect, along with the issue of a rather weak absorption, makes the optimal Yb^3+^ concentration highly dependent on both spectral properties of the particular solar cell type for which this phosphor would be used, as well as parameters of the solar spectrum converting layer itself.

**Figure 11:**
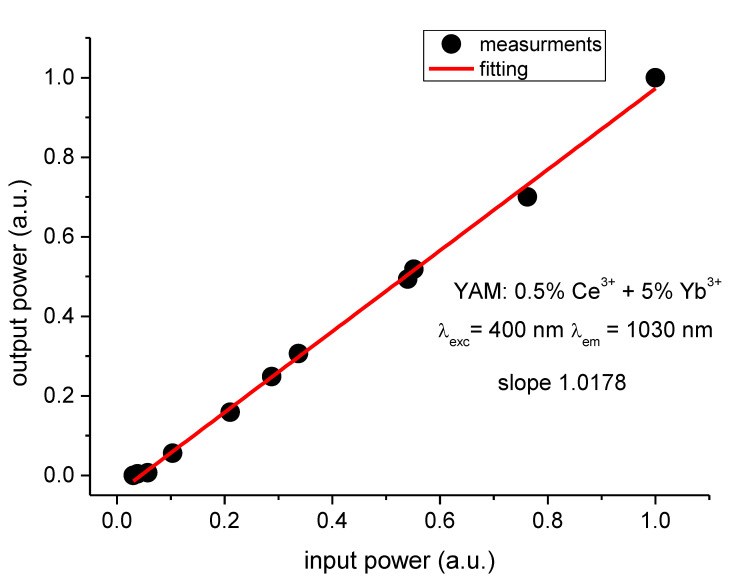
Dependency of Yb^3+^ emission intensity monitored at 1030 nm on excitation power at 400 nm (normalized values).

To further evaluate the actual efficiency of the Ce^3+^ → Yb^3+^ energy transfer, the dependency of Yb^3+^ emission intensity at 1030 nm had been measured in the function of the excitation power of cerium ions at 400 nm, where the excitation spectrum of Yb^3+^ emission has its maximum. The excitation power of the 400 nm laser had been changed using a set of OD filters. A silicon photodiode (sensitive in the 400–1100 nm range), along with a filter cutting off the 400 nm excitation, had been used to monitor the output power. Obtained datapoints were linearly fitted (Figure 11), showing a slope of just 1.0178, which demonstrates that very little, if any, down-conversion occurs and that the energy transfer has a character of down-shifting instead. It should be noted that the observed discrepancy of this result with the efficiencies obtained using decay times analysis is expected, taking into consideration that the latter method gives the maximal value of the energy transfer efficiency.

**Figure 12:**
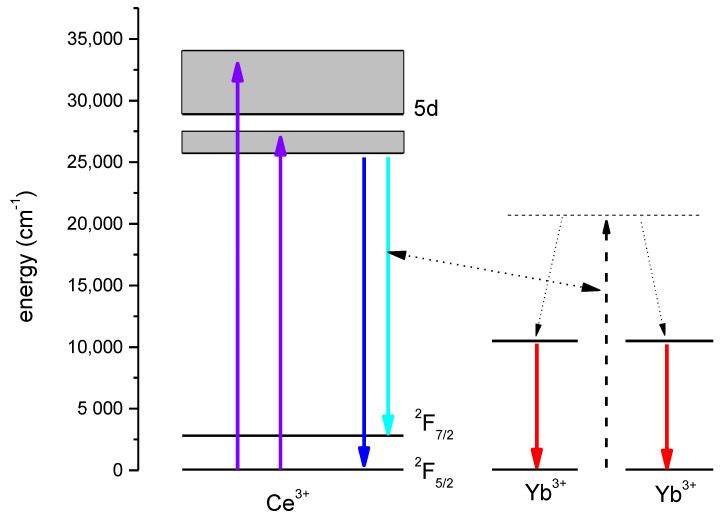
Energy levels of Ce^3+^ and Yb^3+^ in YAG.

The summary of the discussed processes, namely a pathway for obtaining IR emission of Yb^3+^ after the Ce^3+^ excitation in the UV/blue region, is presented in the energy diagram in Figure 12.

## 4. Conclusions

The conducted investigation confirms the existence of a strong energy transfer from Ce^3+^ ions excited in the 350–450 nm range to Yb^3+^ ions, which emit at 970–1070 nm. Further analysis, lifetime, and power ratio measurements suggest that the transfer is a single photon in nature, and not cooperative down-conversion, which would be desirable from application in solar spectrum conversion layers for photovoltaic devices. Figure 10 provides an overview of excitation and emission bands of the YAM:Ce^3+^ + Yb^3+^ system overlapped over standard solar AM1.5G spectrum and typical external quantum efficiency (EQE) of the crystalline silicon solar cell. Optimization of Yb^3+^ ions concentration in the eventual solar spectrum converting layer is difficult, as it not only influences the Yb^3+^ emission in the infrared but also excitation spectra in the visible region. These two effects, however, seem to occur at different concentrations, as the IR emission peak at 980 nm almost disappears at 5% Yb^3+^ concentration, while for this concentration, impact at the excitation spectrum is the greatest among investigated samples. However, excitation and emission bands of Ce^3+^ in YAM are located in a very favorable spectral range from the point of view of downshifting PV applications. As a downshifting layer, this system is not perfect, and eventual benefits would depend highly on the individual solar cell EQE, which denies potential versatility of this system for use in photovoltaics. Optimization of such layers encounters a separate set of problems involving self-absorption of the Yb^3+^ ions’ emissions, a subject discussed extensively in [21]. Potential benefits of application of an idealized down-converting layer are presented in [24].

## Figures and Tables

**Table 1:** List of YAM samples.

No.	Ce^3+^ Concentration (%)	Yb^3+^ Concentration (%)	Remarks
1,2	0.5	0.1	1 mm/min and 5 mm/min pulling speeds
3,4	0.5	1	
5,6	0.5	2	
7,8	0.5	5	
9	0.2	0	absorption meas.
10	0.5	5	reference

## Data Availability

Relevant measurements’ results are presented in the paper, no further datasets are available.
